# Chromosome‐level genome assembly of *Paralithodes platypus* provides insights into evolution and adaptation of king crabs

**DOI:** 10.1111/1755-0998.13266

**Published:** 2020-10-22

**Authors:** Boping Tang, Zhongkai Wang, Qiuning Liu, Zhengfei Wang, Yandong Ren, Huayun Guo, Tingting Qi, Yuetian Li, Huabin Zhang, Senhao Jiang, Baoming Ge, Fujun Xuan, Yue Sun, Shusheng She, Tin Yam Chan, Zhongli Sha, Hui Jiang, Haorong Li, Wei Jiang, Yanli Qin, Kun Wang, Qiang Qiu, Wen Wang, Xinzheng Li, Ngan Kee Ng, Daizhen Zhang, Yongxin Li

**Affiliations:** ^1^ Jiangsu Key Laboratory for Bioresources of Saline Soils Jiangsu Provincial Key Laboratory of Coastal Wetland Bioresources and Environmental Protection Jiangsu Synthetic Innovation Center for Coastal Bio‐agriculture Yancheng Teachers University Yancheng China; ^2^ Center for Ecological and Environmental Sciences Northwestern Polytechnical University Xi’an China; ^3^ Institute of Oceanology Chinese Academy of Sciences Qingdao China; ^4^ Department of Biological Sciences Faculty of Science National University of Singapore Singapore Singapore; ^5^ China Hong Kong Ecology Consultant Company Hong Kong China; ^6^ Institute of Marine Biology National Taiwan Ocean University Keelung Taiwan; ^7^ National Engineering Laboratory of Marine Germplasm Resources Exploration and Utilization Zhejiang Ocean University Zhoushan China; ^8^ National Engineering Research Center for Facilitated Marine Aquaculture Zhejiang Ocean University Zhoushan China

**Keywords:** adaptation, blue king crab, evolution, genome

## Abstract

The blue king crab, *Paralithodes platypus*, which belongs to the family Lithodidae, is a commercially and ecologically important species. However, a high‐quality reference genome for the king crab has not yet been reported. Here, we assembled the first chromosome‐level blue king crab genome, which contains 104 chromosomes and an N50 length of 51.15 Mb. Furthermore, we determined that the large genome size can be attributed to the insertion of long interspersed nuclear elements and long tandem repeats. Genome assembly assessment showed that 96.54% of the assembled transcripts could be aligned to the assembled genome. Phylogenetic analysis showed the blue king crab to have a close relationship with the Eubrachyura crabs, from which it diverged 272.5 million years ago. Population history analyses indicated that the effective population of the blue king crab declined sharply and then gradually increased from the Cretaceous and Neogene periods, respectively. Furthermore, gene families related to developmental pathways, steroid and thyroid hormone synthesis, and inflammatory regulation were expanded in the genome, suggesting that these genes contributed substantially to the environmental adaptation and unique body plan evolution of the blue king crab. The high‐quality reference genome reported here provides a solid molecular basis for further study of the blue king crab's development and environmental adaptation.

## 1 INTRODUCTION

The blue king crab (*Paralithodes platypus*) is the largest crab, even one of the world's largest arthropods, and is routinely commercially caught and eaten. It is protected by a rigid exoskeleton that covers all external parts (Cunningham et al., 1992; Stevens, 2014). The ecologically and commercially valuable blue king crab is widely distributed throughout the North Pacific, including coastal areas of USA, Russia and Japan (Daly & Long, 2014), with combined annual landings of 50,000 metric tons (Otto & Jamieson, 2001). However, its population has declined significantly since the late 1990s (Garber‐Yonts & Lee, 2016). In addition to overfishing, viral‐related diseases have greatly threatened its population health (Ryazanova et al., 2015). Like most marine invertebrates, crustaceans, such as blue king crabs, are exposed to high concentrations of microorganisms. The defence system against microbes in crustaceans is largely dependent on cellular activities, such as adhesion, phagocytosis, encapsulation, nodule formation and melanization, performed by haemocytes. Other antimicrobial factors, such as lysozymes, agglutinins and haemolysins, are also critical parts of their immune system (Stevens, 2014). In addition, the blue king crab has a complex life cycle, with multiple larval stages, including four pelagic larval stages and a semibenthic post‐larval stage (Daly & Long, 2014). During these long larval stages, crabs are particularly vulnerable to pathogens. Although the immune system has been extensively studied (Muraille & Goriely, 2017; Parkin & Cohen, 2001; Takeuchi & Akira, 2010), the genes involved in the immune response of crabs are poorly identified. For example, the *Serpin* gene is known to play a key role in the immune system of many model species (Gettins, 2002; Huntington, 2011; Wang et al., 2017), and its unique immune network has been clearly studied (Dittmann et al., 2015). However, the *Serpin* gene in the king crab is only known to have anticoagulant and anticomplement effects (Dittmann et al., 2015), and other immune responses for adaptative evolution remain to be systematically clarified.

There are 129 king crab species in the 15 genera of the superfamily Lithodoidea (Stevens, 2014). To date, however, only limited RNA‐sequencing (RNA‐seq) data on *Paralithodes camtschaticus* have been released, and no other king crab species, including the blue king crab, have been systematically studied by omics‐based analyses. Based on genome size of crabs in the Animal Genome Size Database (http://genomesize.com), the C‐values of *Necora puber* in Portunidae and *Xantho pilipes* in Xanthidae are 15.17 and 11.77, respectively (Bonnivard et al., 2009), indicating that the genomes of these two crab species are larger than 10 Gb. However, the two published crab genomes, namely swimming crab (*Portunus trituberculatus*) and Chinese mitten crab (*Eriocheir japonica sinensis*), are approximately 1.00 and 1.27 Gb, respectively (Tang, Wang, et al., 2020; Tang, Zhang, et al., 2020). Thus, genome size among crab species appears to vary greatly. Several previous studies have investigated the reason for expanded genome size in specific species, with insertion and replication of transposable elements found to be the primary contributors (Marburger et al., 2018; Naville et al., 2019). Moreover, although all crabs belong to Decapoda, such as *Portunus trituberculatus* and *Eriocheir japonica sinensis*, most of them (called true crabs) belong to Brachyura, but king crabs belong to Anomura. Several studies have analysed the phylogenetic relationships of king crabs, and found that king crabs are closely related to true crabs (Bracken‐Grissom et al., 2013; Tan et al., 2018). However, these studies were based on limited genetic data. Therefore, to better understand various issues related to the king crab, such as genome size, phylogenetic relationship, population history, development and immune response, obtaining a high‐quality reference genome is crucial.

By combining BGI short reads, PacBio long reads and Hi‐C reads, we have generated the first chromosome‐level high‐quality reference genome for the blue king crab, the continuity and completeness of which were comparable with that of other species. Population history analysis revealed that the effective population size declined in the Cretaceous but increased more recently. Gene family analysis identified the expansion of several genes involved in development‐related signalling pathways, steroid and thyroid hormone synthesis, and inflammatory regulation, suggesting that these genes probably contributed to the environmental adaptation of the blue king crab. This high‐quality genome will provide a valuable resource for studying complex development processes and environmental adaptation of the blue king crab.

## 2 MATERIALS AND METHODS

### 2.1 Sampling and sequencing

A male adult blue king crab (*Paralithodes platypus*) was bought from the Sunkfa Company (place of sale: Beidaihe area, China; Figure S1). Genomic DNA was extracted from muscle using a Qiagen Blood & Cell Culture DNA Mini Kit (Qiagen). Three short‐insert‐size libraries with an insert size of ~250 bp were constructed and sequenced on the BGI‐seq 500 platform. A 20‐kb long‐read library was constructed and sequenced on the Sequel platform (Pacific Biosciences). Three Hi‐C libraries were constructed with the restriction endonuclease *Mbo*Ⅰ and sequenced on the BGI‐seq 500 platform. RNAs from four different tissues (liver, gill, stomach and heart) were extracted using a TRIzol kit and sequenced on the BGI‐seq 500 platform.

PacBio long reads were used to construct the skeleton of the genome assembly. BGI‐seq short reads were used to investigate the genome characteristics (such as genome size and heterozygosity) before assembly, as well as for assembly refinement (correcting sequencing errors that exist in long‐reads) and evaluation of assembly quality. After these steps, Hi‐C reads were used to anchor the contig‐level assembly into the final chromosome‐level genome assembly.

### 2.2 Quality control of sequencing data

All sequencing data were filtered to reduce low‐quality bases and duplicated reads using different strategies based on the platforms used. For the BGI‐seq platform data (including genomic short‐reads and RNA‐seq reads), reads were filtered using the following steps: first, PCR duplications in read pairs produced during library construction were removed. Second, adaptors were removed from the sequencing reads. Third, read pairs with more than 50% low‐quality bases were removed. Fourth, read pairs were excluded if any one read had more than 10% unknown bases (Chen et al., 2019). For the PacBio long reads, *subreads* were directly produced by the default parameters of the sequencing equipment (Sequel). For Hi‐C reads, sequencing data were first filtered using the same method as for the BGI‐seq short‐insert reads, then further filtered by hic‐pro (Servant et al., 2015).

### 2.3 Genome size evaluation

To investigate the genome size of the blue king crab, all filtered short‐insert reads from the BGI platform were used for 29‐mer analysis with jellyfish (version 2.2.10; Marcais & Kingsford, 2011). Specifically, reads were artificially divided into 29‐bp length sequences with a 1‐bp step size. If the read length is *L*, the read will produce (*L* − 29 + 1) k‐mers. Therefore, the genome size (*G*) can be estimated by the formula: *G* = *K*_number_/*K*_depth_, where *K*_number_ represents the total number of k‐mers produced and *K*_depth_ represents the peak value of k‐mer depth generated.

### 2.4 Genome assembly

After removing the low‐quality PacBio long‐reads, including low‐score and short reads, all remaining reads were used for genome assembly with wtdbg (https://github.com/ruanjue/wtdbg‐1.2.8). To polish the assembled genome, the PacBio long‐reads were first mapped to the genome assembly by minimap2 (version 2.9; Li, 2017) with default parameters except “‐ax map‐pb,” and then corrected by racon (version 1.2.1; Talay & Altilar, 2009) with four‐round iterations. Because of the high error rate of base‐calling in the PacBio sequencing platform relative to the BGI platform, we further corrected the single‐base errors in the genome assembly using the high‐quality BGI short reads with bwa‐mem (0.7.12‐r1039; Li & Durbin, 2009) for mapping and pilon (version 1.21; Walker et al., 2014) for the correction process (two rounds). Based on the polished assembly and filtered Hi‐C reads, we applied juicer (Durand, et al., 2016) to process the data, then used 3D de novo assembly (version 180419; Dudchenko et al., 2017) to anchor the contigs at the chromosomal level. Lastly, we used juicebox (version 1.9.8; Durand, et al., 2016) to visualize and manually modify the results.

### 2.5 Transcript assembly

Using the clean RNA‐seq data, we de novo assembled the transcripts using bridger (r2014‐12‐01; Chang et al., 2015). These transcripts were further clustered based on pairwise sequence similarity using tgicl (tgicl_linux; Pertea et al., 2003), with the individual clusters then assembled to produce longer, more complete consensus sequences. All transcripts were then used for the evaluation of genome assembly and transcript‐based annotation of protein‐coding genes.

### 2.6 Genome annotation

We annotated repetitive sequences in the blue king crab genome using various software. tandem repeat finder (version 4.04; Benson, 1999) was used to identify tandem repetitive elements, and repeatmodeler was used to de novo detect transposable elements (TEs), including long interspersed nuclear elements (LINEs), short interspersed nuclear elements (SINEs), DNA elements, and long tandem repeats (LTRs). The de novo repeat library produced via repeatmodeler analysis and the RepBase (RepBase16.02) database were separately analysed using repeatmasker (open‐4.0.7; Bedell et al., 2000) to identify repeat sequences. Moreover, we used repeatproteinmask to identify TEs at the protein level. To specifically investigate the main contributors to the large king crab genome, we calculated the insertion times of different TEs based on the formula *T* = *K*/2*R*, where *K* is the Kimura value extracted from the annotation result, *R* is the evolution rate calculated by r8s (Sanderson, 2002) and *T* is the divergence time. Using repeatmasker, the TEs, including LINEs, SINEs, LTRs and DNA elements, were annotated. All annotated TEs were changed to *fasta* format with TE classification as repeatmodeler output consensus sequences. The newly combined consensus sequences were used as the de novo library for repeatmasker annotation and calculation of the Kimura value of each sequence. Divergence times of these species were calculated using r8s. Lastly, we used the Perl script parseRM.pl (downloaded from https://github.com/4ureliek/Parsing‐RepeatMasker‐Outputs) to analyse raw alignment outputs of repeatmasker with the parameters of “‐l 300, 5 ‐age 30 ‐v.”

We then masked all repetitive sequences, except for the tandem repeats, in the blue king crab genome for protein‐coding gene annotation. First, we de novo predicted protein‐coding genes using augustus (version 3.2.1; Stanke & Waack, 2003). Second, protein sequences of eight species, *Drosophila melanogaster* (Ensembl release 95), *Bicyclus anynana* (GCF_900239965.1), *Bombus terrestris* (GCF_000214255.1), *Stegodyphus mimosarum* (GCA_000611955.2), *Penaeus vannamei* (GCA_003789085.1), *Aedes aegypti* (GCF_002204515.2), *Mus musculus* (Ensembl) and *Mesobuthus martensii* (http://lifecenter.sgst.cn/main/en/scorpion.jsp), were downloaded and aligned against the blue king crab genome using tblastn (module in blast) with default parameters except the e‐value was set as 1e‐4. Third, transcripts produced by RNA‐seq reads were translated into amino acids and aligned to the genome using blastn with an e‐value of 1e‐04 and exonerate (version 2.2.0) used for annotation (see Supporting File for detailed parameters). Results from all three methods were integrated using evidencemodeler (version 1.1.1; Haas et al., 2008), with genes exhibiting poor transcript evidence support filtered out. Gene functions were annotated using several databases, including InterPro, SwissProt, TrEMBL, Kyoto Encyclopedia of Genes and Genomes (KEGG) and NCBI nonredundant proteins database (NR). Database alignments for SwissProt, KEGG, TrEMBL and NR were performed using blastp with parameters of “‐e 1e‐05 ‐m 8,” while InterPro annotation was performed using interproscan (version 5.18‐57.0).

### 2.7 Orthologous gene identification

Genes in *Paralithodes platypus* and several other species, including *Drosophila melanogaster*, *Bicyclus anynana*, *Bombus terrestris*, *S. mimosarum*, *Penaeus vannamei*, *A. aegypti*, *Eriocheir japonica sinensis*, *Portunus trituberculatus*, *Daphnia pulex*, *Daphnia magna*, *Eurytemora affinis* and *Hyalella azteca*, were used for relationship determination of homologous genes. The longest transcript of each gene in each species was selected for running all‐to‐all blastp with the parameters “‐evalue 1e‐5 ‐outfmt 6.” The results were then used for relationship determination using orthomcl (version 2.0.9; Li et al., 2003). Finally, 127 genes were found to have only one copy in each species and were thus considered as single‐copy orthologous genes among these species.

### 2.8 Phylogenetic relationships and divergence time estimation

The identified 127 single‐copy orthologous genes among the 13 species (*Drosophila melanogaster*, *Bicyclus anynana*, *Bombus terrestris*, *S. mimosarum*, *Penaeus vannamei*, *A. aegypti*, *Eriocheir japonica sinensis*, *Daphnia pulex*, *Eurytemora affinis*, *H. azteca*, *Daphnia magna*, *Portunus trituberculatus* and *Paralithodes platypus*) by orthomcl (version 2.0.9; Li et al., 2003) were aligned using muscle (version 3.8.31; Edgar, 2004a, 2004b) and concatenated to super‐genes for phylogenetic tree and divergence time analyses. The phylogenetic relationships among species were determined using the concatenated single‐copy genes with the maximum‐likelihood model in raxml (version 8.2.10, see Supporting File for detailed parameters; Stamatakis, 2014). Divergence times among species were analysed using the fourfold degenerate synonymous site with the MCMCtree model in paml (version 4.8; Yang, 1997, 2007), with the fossil records downloaded from the TIMETREE database (www.timetree.org) for calibration of results.

### 2.9 Expansion and contraction analysis of gene family

All identified gene families determined by orthomcl were used as input data in cafe (version 3.1; Han et al., 2013). The phylogenetic relationship results determined by raxml and divergence times determined by mcmctree were also used for expansion/contraction analysis in cafe. To estimate the global error of the input data, we applied an error model before running the cafe program using the python script caferror.py in which the key parameter “lambda ‐s” automatically finds value(s) of lambda that maximize the log likelihood of the data for all families. Estimation of the global error was 6.84e‐04, and the corresponding lambda value, which indicates the probability of both gene gain and loss per gene per unit time in the phylogeny, was 1.57e‐03. Using this lambda value, we applied the main program of cafe for all input gene families as the final step, with these results used for later analyses.

### 2.10 Relative evolution rate

The relative evolution rates of species were analysed by two methods. (a) The concatenated protein sequences were used for analysis by Tajima's relative rate test model in mega7 (Kumar et al., 2016). (b) The *tpcv* model in lintre (Takezaki et al., 1995) was used to test molecular evolution. For both methods, we evaluated the relative evolution rate between the blue king crab and other species, with spider as the outgroup.

### 2.11 Functional enrichment analysis

Genes predicted in the blue king crab genome underwent functional annotation by alignment to the gene ontology (GO) and KEGG databases. Enrichment analysis was performed with gene ID of the blue king crab according to their homologous relationships. GO and KEGG enrichments were analysed by enrichgo, and R scripts, respectively (Beissbarth & Speed, 2004; Huang et al., 2009).

### 2.12 Demographic history reconstruction

The population history of the blue king crab was inferred using the Pairwise Sequentially Markovian Coalescent (PSMC) model. The high‐quality BGI short‐insert‐size reads were aligned to the blue king crab genome by bowtie2 (version 2.2.9; Langmead & Salzberg, 2012), and the alignment results were converted (*samtools view ‐bS*) into *bam* format and sorted (*samtools sort*) using samtools (version 1.3.1; Li et al., 2009). Single nucleotide polymorphisms (SNPs) were identified using bcftools (version 1.3.1; Narasimhan et al., 2016) with key parameters “‐d 150 ‐q 20 ‐Q 20.” The produced results were converted into the input format of PSMC using the vcfutils.pl Perl script (in bcftools). Population history was analysed by PSMC (0.6.5‐r67; Li & Durbin, 2011) using generation time and mutation rate information. PSMC analysis was performed using the parameters “‐N25 ‐r5 ‐p 4 + 25*2 + 4+6,” where ‐N is the maximum number of iterations, ‐r is the initial theta/rho ratio and ‐p is the atomic time intervals. Results were delivered to the plot script using the parameters “‐g 2 ‐u 2.1442e‐9 ‐Y 100,” where ‐g is the number of years per generation and ‐u is the absolute mutation rate per nucleotide per generation. We calculated the mutation rate using r8s (version 1.71) with the calibration time downloaded from the TIMETREE database (www.timetree.org).

## 3 RESULTS

### 3.1 Assembly, annotation and characteristics of the *Paralithodes platypus* genome

To resolve any difficulties that may arise during the genome assembly process, we employed genome survey analysis using ~447.62 Gb short‐insert‐size clean reads produced from the BGI platform (Table S1) before large‐scale sequencing. Evaluation of genome characteristics indicated that the genome was ~5.49 Gb and showed extremely high heterozygosity (Figure S2), thus suggesting a complex genome of blue king crab. To acquire a high‐quality genome assembly, ~229.39 Gb (~41.78‐fold coverage of estimated genome size) PacBio long reads (read number: 24,644,645; average N50: ~15.39 kb) were produced (Table S2). Genome assembly was conducted using wtdbg (version 1.2.8; Ruan & Li, 2020); as a result, we generated a 5.25‐Gb genome assembly with a 154.68‐kb contig N50. Results showed that the size of the assembled blue king crab genome was close to the estimated size. To further improve the quality and accuracy of the genome assembly, we corrected the genome by two steps. First, we employed error correction using all long reads produced on the PacBio platform by racon (version 1.2.1; Talay & Altilar, 2009) with multiple iterations. Second, the racon‐corrected version was further corrected by pilon (version 1.21; Walker et al., 2014) using all BGI‐seq clean data. Finally, we obtained a 4.77‐Gb genome assembly with a 147.47‐kb contig N50 (Table 1). To validate the completeness and accuracy of the blue king crab genome, core genes in Benchmarking Universal Single‐Copy Orthologs (busco; Simao et al., 2015), as well as short reads and transcripts, were used for analysis. In total, more than 225 (74%) and 755 (77%) core eukaryote and metazoan genes, respectively, were successfully identified in the genome (Table 2), and assembly quality was comparable with that of several published species (Table S3). We then aligned all filtered short reads produced on the BGI‐seq platform to the genome using bwa (BWA‐MEM algorithm; Li & Durbin, 2009), with more than 4,106 million reads (97.96%) mapped to the genome (Table S4). Moreover, we de novo assembled the transcripts with the 18.39 Gb of RNA‐seq data using bridger (Chang et al., 2015; Tables S5 and S6). In total, 164,651 transcripts were acquired, with 158,958 (96.54%) mapped to the assembled genome (Table S7). We also found that the average GC content in the blue king crab (~41.57%) was similar to that in other species sampled here (33.62%–42.01%; Figure S3). Until now, no chromosome‐level genome has been reported among Malacostraca species, except for *Portunus trituberculatus*, which is partially because the genome karyotypes of species in these taxa are extremely complex. Thus, construction of a chromosome‐level genome is crucial. Here, ~321.74 Gb of Hi‐C reads (Table S8) were produced for chromosome construction using 3D de novo assembly (Dudchenko et al., 2017). A total of 104 chromosomes were anchored with a mounting rate of 98.80% (Table 3) and with an N50 length of 51.15 Mb (Table S9), suggesting the successful acquisition of a chromosome‐level reference genome for the blue king crab.

**TABLE 1:** Statistics of assembled blue king crab genome

Term	Contig size (bp)	Contig number	Scaffold size (bp)	Scaffold number
N90	26,863	37,023	26,596,420	86
N80	53,963	24,642	34,695,712	71
N70	82,057	17,503	43,689,582	59
N60	112,734	12,546	47,941,768	48
N50	147,467	8,847	51,153,954	39
Max. length (bp)	2,318,639		92,160,374	
Total size (bp)	4,770,095,365		4,805,176,446	
Total number	77,201		6,958	
Average length	61,787		690,597	
Number ≥ 10 kb	57,899		987	

**TABLE 2:** Completeness assessment of the blue king crab genome by busco

Library	Eukaryota	Metazoa
Complete BUSCOs (C)	225	755
Complete and single‐copy BUSCOs (S)	218	738
Complete and duplicated BUSCOs (D)	7	17
Fragmented BUSCOs (F)	45	119
Missing BUSCOs (M)	33	104
Total BUSCO groups searched	303	978
Summary	74.2%	77.2%

**TABLE 3:** Statistics of chromosomal level assembly of *Paralithodes platypus*

Chr ID	Length (bp)	Chr ID	Length (bp)	Chr ID	Length (bp)	Chr ID	Length (bp)
Chr1	32,549,803	Chr27	38,946,284	Chr53	33,834,964	Chr79	93,312,379
Chr2	58,402,507	Chr28	46,543,543	Chr54	36,710,114	Chr80	52,279,954
Chr3	37,676,991	Chr29	51,122,339	Chr55	43,614,431	Chr81	57,170,870
Chr4	51,793,379	Chr30	24,829,886	Chr56	85,512,603	Chr82	45,339,052
Chr5	24,559,915	Chr31	26,693,770	Chr57	59,957,915	Chr83	51,605,793
Chr6	54,221,767	Chr32	49,513,906	Chr58	44,235,702	Chr84	56,625,105
Chr7	23,672,273	Chr33	56,737,213	Chr59	54,921,413	Chr85	68,848,996
Chr8	34,495,329	Chr34	36,334,603	Chr60	76,900,796	Chr86	37,402,623
Chr9	31,797,184	Chr35	29,342,214	Chr61	19,487,293	Chr87	45,508,502
Chr10	33,459,915	Chr36	17,421,013	Chr62	48,531,940	Chr88	32,903,085
Chr11	49,909,878	Chr37	14,583,233	Chr63	9,287,066	Chr89	48,541,041
Chr12	54,897,624	Chr38	18,847,663	Chr64	23,823,223	Chr90	51,617,659
Chr13	39,844,276	Chr39	18,960,315	Chr65	59,670,663	Chr91	50,264,596
Chr14	30,719,803	Chr40	12,196,800	Chr66	45,039,190	Chr92	70,784,578
Chr15	72,452,264	Chr41	33,238,150	Chr67	63,398,095	Chr93	24,684,648
Chr16	79,668,045	Chr42	54,054,138	Chr68	69,107,124	Chr94	59,944,896
Chr17	44,154,361	Chr43	67,509,889	Chr69	65,260,504	Chr95	66,915,750
Chr18	58,690,839	Chr44	13,637,743	Chr70	18,230,472	Chr96	55,462,680
Chr19	61,182,797	Chr45	38,820,040	Chr71	26,928,876	Chr97	32,364,159
Chr20	23,765,179	Chr46	56,444,004	Chr72	44,271,896	Chr98	35,129,409
Chr21	49,620,838	Chr47	34,247,924	Chr73	37,049,393	Chr99	32,926,917
Chr22	34,159,061	Chr48	48,416,605	Chr74	71,913,056	Chr100	63,007,124
Chr23	42,192,145	Chr49	47,013,507	Chr75	13,577,550	Chr101	22,827,927
Chr24	32,794,045	Chr50	66,020,221	Chr76	74,513,614	Chr102	58,963,391
Chr25	47,468,639	Chr51	44,537,028	Chr77	49,383,150	Chr103	59,497,576
Chr26	59,426,035	Chr52	53,295,507	Chr78	63,263,219	Chr104	72,347,307
Total chromosome‐level contig length				4,747,582,709			
Total contig length				4,805,176,446			
Chromosome length/total length				98.80%			

### 3.2 Rapid outbreak of repetitive sequences contributed to large *Paralithodes platypus* genome

**FIGURE 1:**
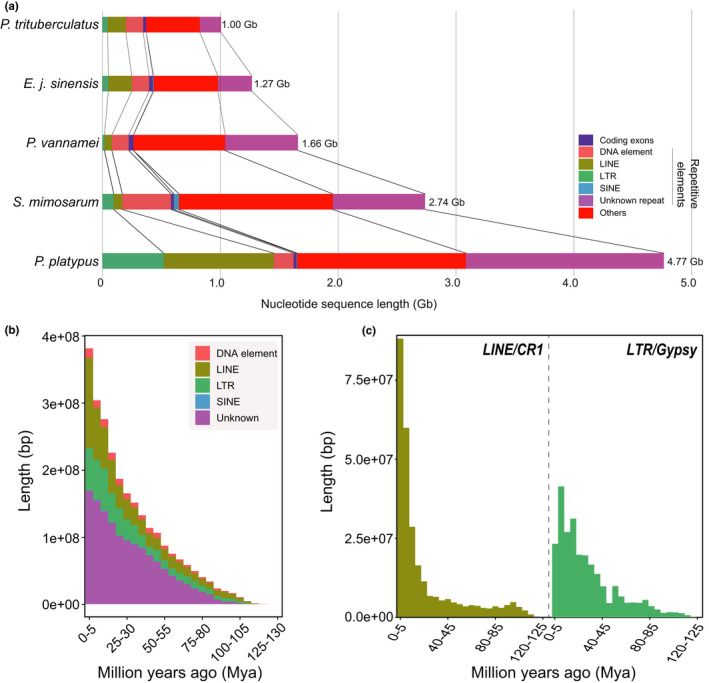
Outbreak of a large number of repetitive sequences in the blue king crab genome. (a) Composition of whole‐genome assemblies into different segments in three species. “Unknown repeat” refers to other types of repetitive sequences, excluding LINE, SINE, LTR and DNA elements. “Others” refers to sequences not predicted as coding or repetitive sequences. (b) Outbreak/insertion history of repetitive sequences in the blue king crab genome. (c) Two specific subtypes in the blue king crab genome [Colour figure can be viewed at wileyonlinelibrary.com]

The blue king crab has a large genome (4.77 Gb) relative to several species, including the swimming crab (1.00 Gb; 4.77‐fold), Chinese mitten crab (1.27 Gb; 3.76‐fold), shrimp (1.66 Gb; 2.87‐fold) and spider (2.74 Gb; 1.74‐fold; Figure 1a). Therefore, to study genome expansion, we performed genome annotations in the blue king crab. We first annotated repetitive sequences in the blue king crab genome by both the de novo and homologue methods, resulting in the identification of ~3.71 Gb of repeat sequences, accounting for 77.73% of the assembled blue king crab genome (Table S10). Among these repetitive sequences, LINEs accounted for the highest proportion (percentage: 19.59%; total length: 934.36 Mb) in the genome among all known transposable elements (e.g., LTRs, DNA elements, LINEs, SINEs), followed by LTRs (percentage: 10.92%; total length: 520.71 Mb) and DNA elements (percentage: 3.51%; total length: 167.40 Mb). The SINE content (percentage: 0.22%; total length: 10.69 Mb) accounted for the lowest proportion among the four types of repetitive sequences (Figure 1a; Table 4). To clarify whether coding regions also contributed to the large blue king crab genome, we compared them with other species. However, we found no significant differences in the total length of coding regions among these genomes, suggesting that the coding regions did not play a key role in the expansion of the blue king crab genome. We next investigated the expansion ratio of repetitive elements in the blue king crab genome relative to the above four species. Results showed that LINE repetitive elements in the king crab genome were more than four‐fold higher that of the other species (swimming crab: 6.11‐fold; Chinese mitten crab: 4.64‐fold; shrimp: 14.82‐fold; spider: 12.79‐fold), and LTR (swimming crab: 11.52‐fold; Chinese mitten crab: 10.47‐fold; shrimp: 29.37‐fold; spider: 5.40‐fold), SINE (swimming crab: 74.84‐fold; Chinese mitten crab: 2.32‐fold; shrimp: 9.24‐fold; spider: 0.25‐fold) and DNA elements (swimming crab: 1.12‐fold; Chinese mitten crab: 1.12‐fold; shrimp: 1.17‐fold; spider: 0.40‐fold) exhibited various degrees of change (Figure 1a). These results suggest that the explosion of LINE and LTR, but not SINE and DNA elements, greatly contributed to the evolution of the large blue king crab genome. To further investigate the LINE and LTR types that contributed to the large genome, we annotated their small types and found that CR1 (length: 276.21 Mb; percentage in LINE: 29.56%) and Gypsy (length: 255.07 Mb; percentage in LTR: 48.99%) contributed most to LINE and LTR types, respectively (Table S11). We further investigated the expansion history of repetitive elements and found that the blue king crab experienced one expansion event within the last ~100 million years (Figure 1b), with both Gypsy and CR1 showing a sharp expansion within the most recent ~40 and ~20 million years, respectively (Figure 1c). Therefore, these results indicate that LINE and LTR expansions were major contributors to the large genome size in the blue king crab, with expansion history and time shown in Figure 1.

**TABLE 4:** Statistics of de novo annotated repeat sequences in the blue king crab genome

Type	Length (bp)	Percentage in genome (%)
DNA	167,399,766	3.509359
LINE	934,357,039	19.587806
SINE	10,692,924	0.224166
LTR	520,710,193	10.916138
Other	27,431,494	0.005750722
Satellite	2,029,331	0.042543
Simple repeat	226,434,823	4.746966
Unknown	1,499,411,342	31.433571
Total	3,312,560,085	69.444315

### 3.3 Phylogenetic relationship and evolutionary history of blue king crab based on comparative genomic analysis

**FIGURE 2:**
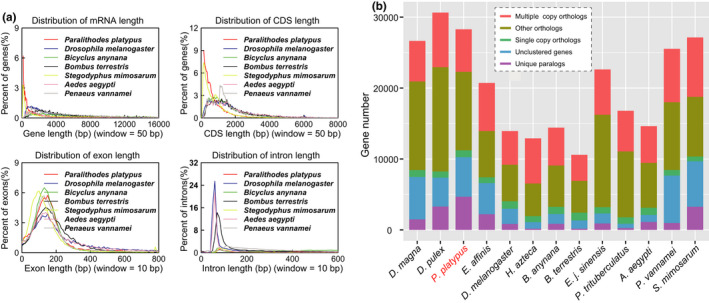
Protein‐coding gene annotation and orthologous gene analysis among species. (a) Quality of gene annotation shown with gene length, CDS length, exon length and intron length, respectively. (b) Number of orthologous genes among species [Colour figure can be viewed at wileyonlinelibrary.com]

**FIGURE 3:**
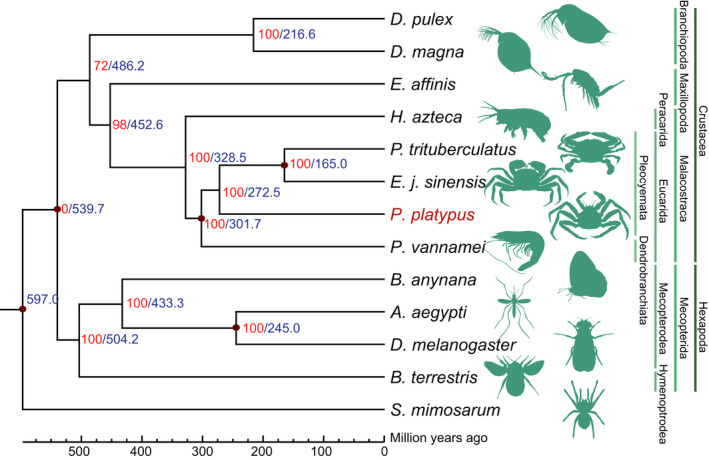
Phylogenetic relationship and divergence time among species. Blue numbers on branches represent the divergence time between two branches and red number at each node represents the bootstrap value. Dark red circles represent the time of the fossil record used [Colour figure can be viewed at wileyonlinelibrary.com]

**FIGURE 4:**
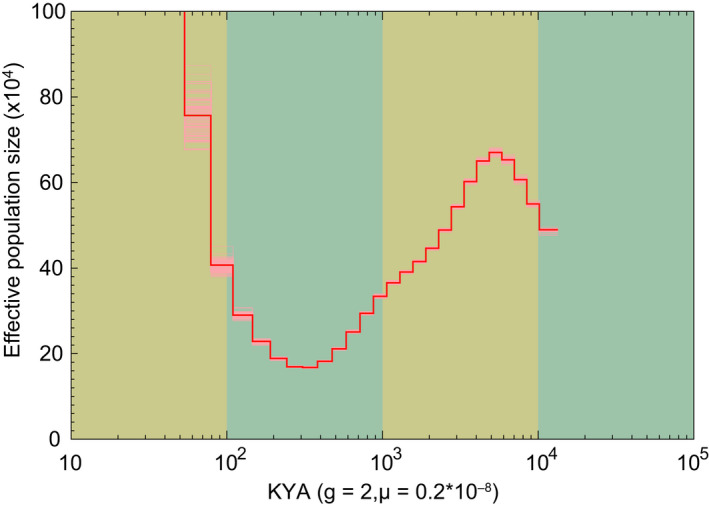
Population history analysis of the blue king crab. This figure reflects changes in effective population size over time. Parameter “g” represents generation time, parameter “μ” represents mutation rate per generation and KYA represents thousand years ago [Colour figure can be viewed at wileyonlinelibrary.com]

**FIGURE 5:**
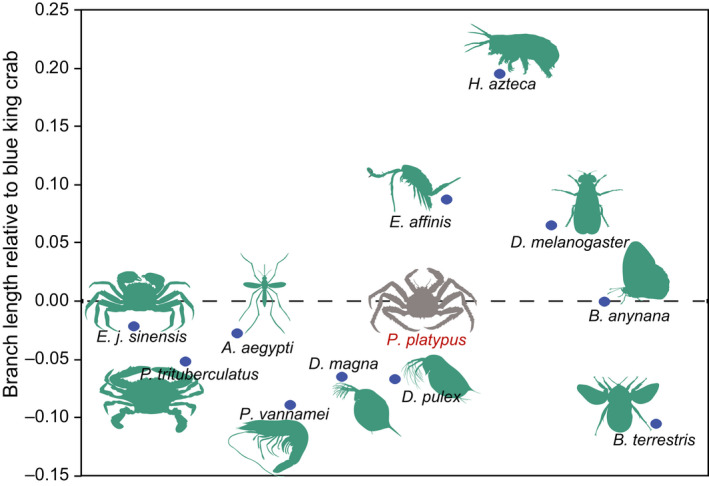
Relative evolution rate analysis among species. The *x*‐axis shows species used in this study; the *y*‐axis shows relative branch lengths of each species to the blue king crab. Blue king crab was used as the reference species; spider was used as the outgroup species [Colour figure can be viewed at wileyonlinelibrary.com]

We used protein‐coding gene annotation and identified 28,287 high‐quality protein‐coding genes. To check the prediction quality of the protein‐coding genes, we compared mRNA, coding sequence (CDS), exon and intron lengths and found that the quality of the predicted genes in the blue king crab was comparable with that of other species examined (Figure 2a). Gene functional annotation was employed using the InterPro, SwissProt, TrEMBL, GO, KEGG and NR databases. Results showed that most of the predicted genes had functional annotations in these databases (Table 5). Using comparative genomics, the gene families among 13 species (*Drosophila melanogaster*, *Bicyclus anynana*, *Bombus terrestris*, *S. mimosarum*, *Penaeus vannamei*, *A. aegypti*, *Eriocheir japonica sinensis*, *Daphnia pulex*, *Eurytemora affinis*, *H. azteca*, *Daphnia magna*, *Portunus trituberculatus*, and *Paralithodes platypus*) were analysed. The longest transcript of each individual gene was selected and aligned to each other using blastp and clustered using orthomcl (Li et al., 2003). As a result, 15,990 gene families were constructed, with 127 single‐copy genes identified among the 13 species (Figure 2b; Table S12). Previous nuclear gene studies have indicated that the blue king crab has a very close phylogenetic relationship with true crabs (Bracken‐Grissom et al., 2013; Tan et al., 2018). However, whether whole‐genome data show the same results remains unclear. Here, we aligned all single‐copy genes among the 13 species using muscle (version 3.8.31; Edgar, 2004a, 2004b), with protein alignments, and then concatenated to supergenes for phylogenetic analysis by raxml (‐m PROTGAMMAAUTO; version 8.2.10; Stamatakis, 2014) and with spider (*S. mimosarum*) as an outgroup. We found that the blue king crab indeed showed the closest relationship with two true crabs, that is Chinese mitten crab (*Eriocheir japonica sinensis*) and swimming crab (*Portunus trituberculatus*), followed by shrimp (*Penaeus vannamei*), and then *H. azteca*, suggesting a close evolutionary relationship between blue king crab and the other species, which all belong to Malacostraca (Figure 3). In addition, the 12 species of pan‐crustaceans (*Daphnia pulex*, *Daphnia magna*, *Eurytemora affinis*, *H. azteca*, *Penaeus vannamei*, *Paralithodes platypus*, *Eriocheir japonica sinensis*, *Portunus trituberculatus*, *Bombus terrestris*, *Bicyclus anynana*, *A. aegypti* and *Drosophila melanogaster*) formed two clades: Hexapoda and Crustacea. The Hexapoda group consisted of all Hymenoptera (*Bombus terrestris*), Lepidoptera (*Bicyclus anynana*) and Diptera (*A. aegypti* and *Drosophila melanogaster*) insects, with Diptera and Lepidoptera forming the Mecopterodea clade (*Bicyclus anynana*, *A. aegypti* and *Drosophila melanogaster*) followed by the Hymenoptera insect (*Bombus terrestris*). The Crustacea group comprised all crustaceans, with *Paralithodes platypus* and Brachyura (*Portunus trituberculatus* and *Eriocheir japonica sinensis*) forming a Pleocyemata clade, followed by the Dendrobranchiata shrimp (*Penaeus vannamei*). The two clades were united in a monophyletic group named Eucarida, which is the sister clade to Percarda in class Malacostraca. Branchiopoda (*Daphnia pulex* and *Daphnia magna*), Maxillopoda (*Eurytemora affinis*) and Malacostraca (*H. azteca*, *Portunus trituberculatus*, *Eriocheir japonica sinensis*, *Paralithodes platypus* and *Penaeus vannamei*) formed the Crustacea clade, and Hexapoda and Crustacea were each monophyletic (Figure 3). The divergence time between the blue king crab and true crabs remains unclear, although previous study using two mitochondrial genes and three nuclear genes indicates that the king crab diverged from the ancestor of true crabs ~259 million years ago (Ma; Bracken‐Grissom et al., 2013). To further investigate and determine the divergence time of the blue king crab and true crabs, we used all single‐copy genes among the 13 species for analysis by mcmctree (in paml; Yang, 1997, 2007). Results indicated that the blue king crab and two true crabs (i.e., Chinese mitten crab and swimming crab) diverged ~272.5 Ma. Furthermore, these three crabs diverged from shrimp ~301.7 Ma, Peracarida (*H. azteca*) diverged from Eucarida 328.5 Ma, and Malacostraca diverged from Maxillopoda 452.6 Ma (Figure 3). Population history analysis indicated that the effective population size of the blue king crab declined ~1 Ma, but then began to expand ~100,000 years ago, suggesting a possible increase in genetic diversity and adaptation (Figure 4). Relative evolution rate analysis can clarify the historical status and the rate of molecular evolution of a species. We analysed the blue king crab and other examined species and found that the blue king crab has a relatively faster evolution rate than the swimming crab and Chinese mitten crab (Figure 5; Tables S13 and S14).

**TABLE 5:** Statistics of functional annotation of protein‐coding genes in the blue king crab genome

Database	Annotated gene number	Percentage (%)
InterPro	11,782	41.65
GO	8,284	29.29
KEGG	9,315	32.93
SwissProt	10,733	37.94
TrEMBL	15,540	54.94
NR	16,151	57.10

### 3.4 Specific/expanded gene families facilitate development of unique features and physiology

**FIGURE 6:**
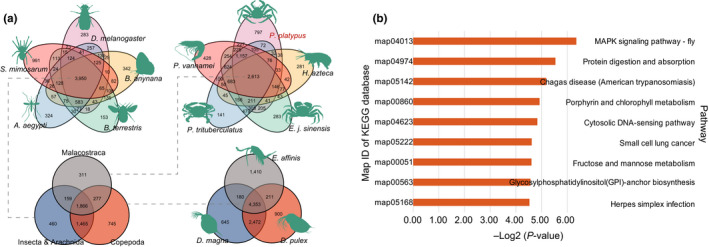
Gene family analysis among species. (a) Number of shared and unique gene families in species. (b) Enrichment analysis of specific gene families in the blue king crab relative to other Malacostraca species. The *y*‐axis shows significantly enriched biological pathways [Colour figure can be viewed at wileyonlinelibrary.com]

Using the clustering results of these species, we performed gene family analysis. We found 1,866 gene families that commonly existed in the 13 species (Figure 6a). In addition, 2,613 gene families existed in all five Malacostraca species (*H. azteca*, *Portunus trituberculatus*, *Eriocheir japonica sinensis*, *Paralithodes platypus* and *Penaeus vannamei*; Figure 6a), and 20 GO terms, such as carbohydrate metabolic process (*p* = 6.45e‐04) and primary metabolic process (*p* = 2.11e‐04), and 86 KEGG terms, such as glutathione metabolism (*p* = 6.10e‐06), retinol metabolism (*p* = 4.15e‐04), sphingolipid metabolism (*p* = 9.17e‐05) and fatty acid metabolism (*p* = 1.32e‐02), were functionally enriched (Tables S15 and S16). Among the five Malacostraca species, we identified 797 unique gene families in *Paralithodes platypus*, 283 in *Eriocheir japonica sinensis*, 428 in *Penaeus vannamei*, 141 in *Portunus trituberculatus* and 281 in *H. azteca*. Further enrichment analysis demonstrated that the unique gene families in the blue king crab were mainly involved in the MAPK signalling pathway (*p* = 1.22e‐02), protein digestion and absorption (*p* = 2.15e‐02), cytosolic DNA‐sensing pathway (*p* = 3.50e‐02), fructose and mannose metabolism (*p* = 4.09e‐02), glycosylphosphatidylinositol (GPI)‐anchor biosynthesis (*p* = 4.21e‐02) and herpes simplex infection (*p* = 4.32e‐02), suggesting that genes in these families may play key roles in the biosynthesis and metabolism processes of its unique body plan as well as in physiological processes (Figure 6b; Tables S17 and S18).

**FIGURE 7:**
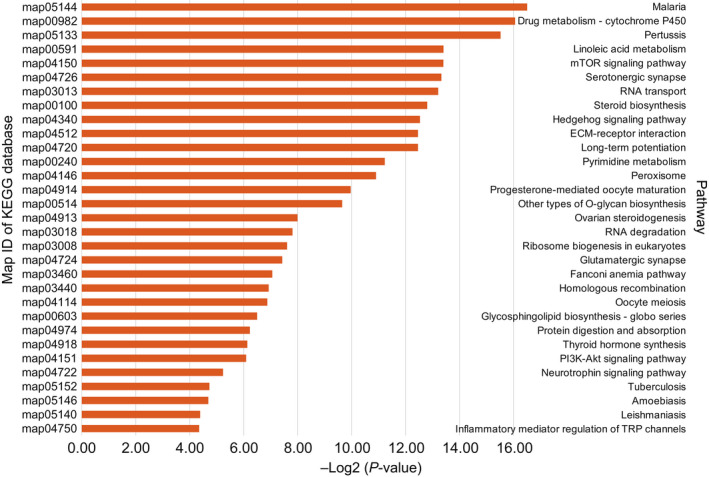
Functional enrichment of expanded gene families in the blue king crab. The *x*‐axis shows negative logarithm of the *p*‐value; the *y*‐axis shows significantly enriched biological pathways [Colour figure can be viewed at wileyonlinelibrary.com]

To investigate the expanded gene families in the above species, we employed expansion and contraction analysis of gene families using cafe (Han et al., 2013), and found that several gene families were changed in the five Malacostraca species (Tables S19–S22). We further studied whether the blue king crab showed unique changes in gene families and identified 98 expanded gene families and 113 contracted gene families in this species (*p* < .01). Functional enrichment analysis of these families (Tables S23–S26) indicated that, among the expanded gene families, 20 GO and 31 KEGG pathway terms were significantly enriched (*p* < .05). Further examination of the expanded gene families demonstrated that they were mainly related to biological processes, such as the mTOR signalling pathway (*p* = 9.24e‐05), Hedgehog signalling pathway (*p* = 1.69e‐04), PI3K‐Akt signalling pathway (*p* = 1.47e‐02), steroid biosynthesis (*p* = 1.40e‐04), thyroid hormone synthesis (*p* = 1.42e‐02), homologous recombination (*p* = 8.22e‐03) and inflammatory mediator regulation of TRP channels (*p* = 4.91e‐02; Figure 7; Tables S23 and S24). The mTOR, Hedgehog and PI3K‐Akt signalling pathways play several critical roles in development and other physiological processes. Together with inflammatory regulation and homologous recombination, our results suggest that these biological pathways may be related to development and immune defence processes in the blue king crab.

## 4 DISCUSSION

**FIGURE 8:**
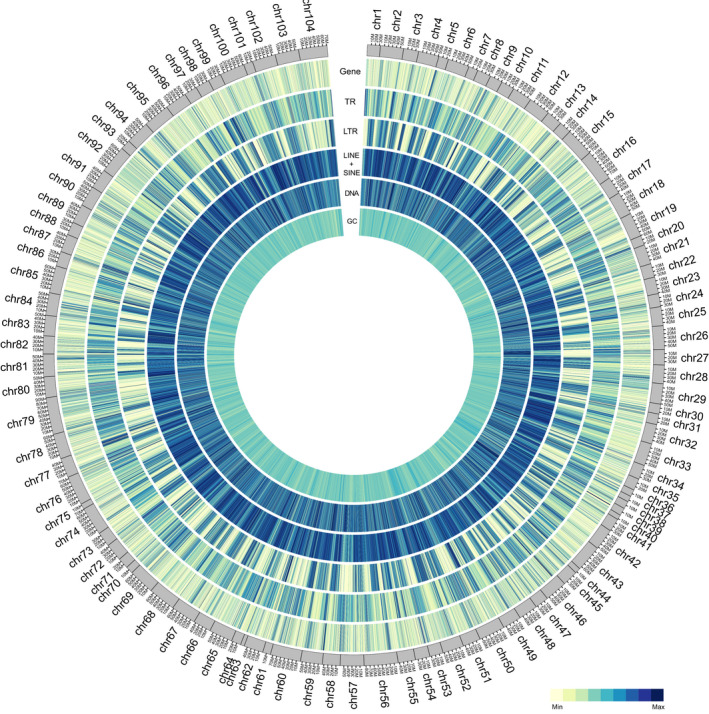
Blue king crab genome assembly, annotation and characteristics. Tracks A to F represent the distribution density of protein‐coding genes, tandem repeats, long tandem repeats, long/short interspersed nuclear elements, DNA elements and GC, respectively, with density calculated in 0.2‐Mb windows [Colour figure can be viewed at wileyonlinelibrary.com]

The blue king crab has great economic value and occupies an important evolutionary position. Genomic information regarding this species could help better understand its rapid reproductive rate as well as its unique body plan and environmental adaptation. Here, based on various sequencing platforms, we generated different sequencing data, including ~447.62 Gb of BGI‐seq short‐insert reads, ~229.39 Gb of PacBio long reads, ~321.74 Gb of Hi‐C sequencing reads and ~18.39 Gb of transcriptomic data, to construct and annotate a chromosome‐level reference genome of the blue king crab. Using a hybrid assembly method combining wtdbg, racon, pilon and 3D de novo assembly, we successfully acquired a 4.77‐Gb chromosome‐level genome assembly of the blue king crab, which represents the first reference genome of Anomura (Figure 8; Tables 1 and 3; Tables S1, S2, S5 and S8). Genomic comparisons revealed this assembly to be the most complex chromosome‐level genome assembly in Malacostraca to date, with successful anchoring of 104 chromosome sequences (Figure 8; Table 3). This number is consistent with chromosome results from karyotype analysis of another king crab species (*Paralithodes camtschatica*; Niiyama, 1935), further supporting the accurate chromosome number acquired in the current study. Therefore, this genome assembly could act as a reference genome for studies on Malacostraca species. Our results also showed that the blue king crab had a large genome relative to the other arthropods examined. The main reason for genome expansion in the blue king crab was the expansion of LINE and LTR repetitive elements (Figure 1a,b). Further analysis indicated that CR1 and Gypsy accounted for the largest proportions in LINE and LTR types and sharply increased within the last ~20 and ~40 million years, respectively (Figure 1c). These analyses will assist in our understanding of the expansion history of the large blue king crab genome.

Phylogenetic analysis using various high‐quality genome assemblies of Crustacea and several other representative species showed that the blue king crab has a close relationship with true crabs, including the Chinese mitten crab and swimming crab, with divergence ~272.5 Ma (Figure 2a). Gene family analysis identified several expanded gene families in blue king crab, including those related to several signalling pathways (mTOR, Hedgehog and PI3K‐Akt signalling pathways), biosynthesis processes (steroid biosynthesis and thyroid hormone synthesis), inflammatory reaction and homologous recombination (Figure 7; Table S24). These signalling pathways, especially the Hedgehog pathway, play several key conserved roles in early development and body plan formation of many species (Chuong et al., 2000). For example, previous studies of mice and zebrafish indicate that the Hedgehog pathway plays a key role in craniofacial development (Schwend & Ahlgren, 2009). In Drosophila, the Hedgehog protein regulates development of the eye imaginal disc (Strutt & Mlodzik, 1996). In mammals, the Hedgehog signalling pathway plays a central role in gonadal (and thus sexual) development (Barsoum, 2009). Our results together with those of previous studies further suggest that related genes may have contributed to the development and formation of the unique body plan of the king crab. The blue king crab has a long and complex development process and is vulnerable to virus attack during its lengthy larval stages. Interestingly, our comparative genomic analysis found that gene families involved in inflammatory reactions were expanded in the blue king crab, which may partly explain the strong environmental adaptation ability of king crabs. Like marine invertebrates, and indeed all marine animals, blue king crabs are constantly exposed to high concentrations of microorganisms. However, they lack the elements of adaptive immunity, such as T‐cells and B‐cells, and primarily depend on other immune factors, such as antimicrobial peptides, to combat invading pathogens (Moe et al., 2018). Therefore, the expansion of immune‐related genes may help king crabs avoid injury and invasion from pathogens, and thus better adapt to the marine environment. We also found that related homologous recombination genes were expanded in the king crab, suggesting that this species could quickly repair damaged genomic DNA via homologous recombination. Together, the genome assembly and transcriptomic data generated in this study will provide valuable resources for research on the developmental processes and adaptive evolution of the blue king crab.

## AUTHOR CONTRIBUTIONS

Y.L., D.Z., N.N. and X.L. conceived the project. B.T., D.Z., Q.L., S.J., Z.W., F.X., B.G., H.Z., W.J., Y.S., H.G., T.Q., Yuetian L., N.N., S.S. and X.L. collected and dissected the samples. Z.W., H.J. and Y.L. estimated the genome size. Y.L. assembled the genome. Z.W., Y.R., Y.L., H.L. and Y.Q. performed the genome assembly, genome annotation and evolutionary analyses. Y.L., Q.Q., W.W. and B.T. wrote the manuscript. K.W., W.W., Q.Q., T.C., Z.S. and B.T. revised the manuscript.

## Supporting information

Supplementary Material

## Data Availability

Raw sequencing data and the genome assembly have been deposited in the National Center for Biotechnology Information (NCBI) database with accession no. PRJNA555178. The genome assembly has also been deposited in the Dryad database (https://datadryad.org/stash/dataset/doi:10.5061/dryad.jm63xsj6c).
