# Supplementary material for: Chromosome‐level genome assembly of Paralithodes platypus provides insights into evolution and adaptation of king crabs
Source: Mol Ecol Resour. 2020 Oct 22;21(2):511–25. doi: 10.1111/1755-0998.13266 (PMC7821229; doi:10.1111/1755-0998.13266)
Supplement: Supplementary file 1 — Supplementary Material [file MEN-21-511-s001.docx]

**Table S1. Statistics of clean short-reads generated using BGI-seq 500 platform.**

| Library | Total bases (PE) | Sequencing strategy | Sequencing depth (X) |
| --- | --- | --- | --- |
| L1 | 145,702,080,304 | PE100 | 26.54 |
| L2 | 151,301,245,028 | PE100 | 27.56 |
| L3 | 150,612,321,134 | PE100 | 27.43 |
| Total | 447,615,646,466 | - | 81.53 |

Note: The sequencing depth was calculated with the estimated genome size.

**Table S2. Statistics of long-reads generated using PacBio Sequel platform.**

| Cell ID | Total bases | Total reads number | Max length (bp) | Average length (bp) | N50 length (bp) |
| --- | --- | --- | --- | --- | --- |
| m54136_180731_085011 | 7,215,429,940 | 831,530 | 85,455 | 8,677 | 14,921 |
| m54136_180801_103725 | 6,923,276,027 | 796,247 | 76,206 | 8,694 | 14,719 |
| m54136_180801_205028 | 6,325,375,586 | 806,561 | 76,202 | 7,842 | 13,551 |
| m54136_180802_070641 | 7,187,346,247 | 788,860 | 82,612 | 9,111 | 15,421 |
| m54136_180804_101006 | 8,165,772,561 | 831,272 | 79,017 | 9,823 | 15,836 |
| m54136_180807_191740 | 9,034,458,335 | 951,691 | 81,132 | 9,493 | 15,774 |
| m54136_180808_052954 | 7,245,638,649 | 844,889 | 88,258 | 8,575 | 14,654 |
| m54136_180814_100433 | 7,976,547,061 | 813,490 | 83,877 | 9,805 | 15,853 |
| m54136_180815_200030 | 7,152,093,234 | 753,264 | 74,831 | 9,494 | 15,441 |
| m54136_180816_061553 | 7,635,620,524 | 726,921 | 93,837 | 10,504 | 16,299 |
| m54136_180816_163214 | 7,551,311,227 | 775,126 | 76,815 | 9,742 | 15,685 |
| m54136_180818_055946 | 7,562,258,606 | 754,710 | 83,540 | 10,020 | 16,038 |
| m54136_180818_161654 | 7,055,296,314 | 739,661 | 72,199 | 9,538 | 15,387 |
| m54136_180819_090957 | 6,274,369,250 | 662,999 | 82,022 | 9,463 | 15,362 |
| m54136_180820_155845 | 7,044,514,261 | 670,857 | 81,371 | 10,500 | 16,464 |
| m54136_180821_172628 | 6,649,436,473 | 674,001 | 79,336 | 9,865 | 15,805 |
| m54136_180822_034246 | 6,475,567,492 | 652,254 | 80,754 | 9,927 | 15,884 |
| m54136_180822_135839 | 6,597,722,965 | 716,574 | 73,189 | 9,207 | 15,117 |
| m54136_180823_041748 | 7,383,527,179 | 773,758 | 78,938 | 9,542 | 15,595 |
| m54136_180823_143219 | 7,206,380,087 | 748,359 | 88,766 | 9,629 | 15,631 |
| m54136_180824_005016 | 6,456,341,342 | 710,328 | 89,128 | 9,089 | 14,868 |
| m54139_180728_091624 | 7,436,522,134 | 828,063 | 82,641 | 8,980 | 15,134 |
| m54139_180803_073931 | 8,247,646,019 | 884,398 | 91,645 | 9,325 | 15,648 |
| m54139_180803_175352 | 7,942,265,569 | 862,998 | 89,753 | 9,203 | 15,522 |
| m54139_180804_083645 | 8,208,714,354 | 899,968 | 82,700 | 9,121 | 15,681 |
| m54139_180805_051556 | 3,965,443,761 | 507,657 | 87,473 | 7,811 | 13,916 |
| m54139_180805_153114 | 7,535,412,985 | 878,517 | 78,509 | 8,577 | 14,856 |
| m54139_180806_072743 | 7,256,175,284 | 846,431 | 79,285 | 8,572 | 14,805 |
| m54139_180806_174331 | 7,632,911,510 | 870,670 | 76,807 | 8,766 | 14,999 |
| m54139_180818_122743 | 6,271,493,342 | 682,451 | 72,291 | 9,189 | 15,302 |
| m54139_180820_085407 | 6,940,422,262 | 692,711 | 79,861 | 10,019 | 15,975 |
| m54139_180820_190623 | 6,831,387,000 | 667,429 | 83,368 | 10,235 | 16,181 |
| Total/Average | 229,386,677,580 | 24,644,645 | - | 9,323 | 15,385 |

**Table S3. Genome quality statistics of available closely related species with *P. platypus*.**

| Species | Complete BUSCO (%) | Fragmented BUSCO (%) | Missing BUSCO (%) | BUSCO dataset |
| --- | --- | --- | --- | --- |
| *P. platypus* | 74.2 | 14.9 | 10.9 | eukaryota |
| *M. martensii* | 58.4 | 27.1 | 14.5 | eukaryota |
| *E. j. sinensis* | 76.3 | 4.3 | 19.4 | eukaryota |

**Table S4. Statistics of short read mapping ratio of assembled genome.**

| Term | Number | | Percent (%) |
| --- | --- | --- | --- |
| Mapped reads | 4,106,160,511 | | 97.96 |
| Properly paired mapped reads | 3,643,435,638 | | 87.99 |
| Total reads | 4,191,875,222 | 100.00 | |

**Table S5. Statistics of clean RNA-seq reads generated using BGI-seq 500 platform.**

| Term | Total reads (PE) | Total bases (PE) |
| --- | --- | --- |
| Liver | 26,440,716 | 4,759,328,880 |
| Gill | 27,301,041 | 4,914,187,380 |
| Stomach | 25,454,716 | 4,581,848,880 |
| Heart | 22,970,289 | 4,134,652,020 |
| Total | 102,166,762 | 18,390,017,160 |

**Table S6. Statistics of assembled transcripts by Bridger and TGICL softwares in *P. platypus*.**

| Term | Size (bp) | Number |
| --- | --- | --- |
| N90 | 658 | 12,901 |
| N80 | 957 | 9,530 |
| N70 | 1,278 | 7,110 |
| N60 | 1,640 | 5,254 |
| N50 | 2,044 | 3,786 |
| Max length (bp) | 16,104 | |
| Total size (bp) | 26,867,094 | |
| Total number | 18,871 | |
| Average length | 1,423 | |
| Number>=1000bp | 9,148 | |

**Table S7. Statistics of transcript mapping ratio on assembled genome.**

| Range of length | Total number | Total match number | Percent  (%) | >50% of sequence | | >90% of sequence | |
| --- | --- | --- | --- | --- | --- | --- | --- |
|  |  |  |  | Number | Percent  (%) | Number | Percent  (%) |
| All | 164,651 | 158,958 | 96.54 | 140,964 | 85.61 | 92,962 | 56.46 |
| ≥500 | 65,030 | 51,444 | 79.11 | 55,699 | 85.65 | 28,885 | 44.42 |
| ≥1000 | 28,996 | 20,201 | 69.67 | 24,009 | 82.80 | 10,835 | 37.37 |

**Table S8. Statistics of clean Hi-C reads generated using BGI-seq 500 platform.**

| Library | Total reads (PE) | Total bases (PE) | Sequencing depth (X) |
| --- | --- | --- | --- |
| L1 | 1,220,343,120 | 122,034,312,000 | 22.23 |
| L2 | 1,152,652,704 | 115,265,270,400 | 21.00 |
| L3 | 844,360,510 | 84,436,051,000 | 15.38 |
| Total | 3,217,356,334 | 321,735,633,400 | 58.61 |

Note: The sequencing depth was calculated with the estimated genome size.

**Table S9. Genome assembly statistics of available closely related species with *P. platypus*.**

| Species | Genome size | N50 | Assembly level |
| --- | --- | --- | --- |
| *Paralithodes platypus* | 4,805,176,446 | 51,153,954 | chromosome |
| *Mesobuthus martensii* | 925,546,267 | 45,228 | scaffold |
| *Drosophila melanogaster* | 143,726,002 | 25,286,936 | chromosome |
| *Stegodyphus mimosarum* | 2,738,704,917 | 480,636 | chromosome |
| *Bombus terrestris* | 248,654,244 | 12,868,931 | chromosome |
| *Bicyclus anynana* | 475,399,557 | 638,282 | scaffold |
| *Penaeus vannamei* | 1,663,565,311 | 605,555 | scaffold |
| *Eriocheir japonica sinensis* | 1,118,179,523 | 111,755 | scaffold |

**Table S10. Statistics of annotated repetitive sequences in *P. platypus* genome.**

| Type | Repeat Size (bp) | % of genome |
| --- | --- | --- |
| Trf | 610,173,583 | 12.79 |
| Repeatmasker | 325,922,674 | 6.83 |
| Proteinmask | 878,649,676 | 18.42 |
| *De novo* | 3,312,560,085 | 69.44 |
| Total | 3,707,846,984 | 77.73 |

**Table S11. Composition of main repetitive sequences in *P. platypus* genome.**

| Class | Type | Length (bp) | Percent (%) |
| --- | --- | --- | --- |
| DNA | DNA/Academ | 27,075,612 | 0.56 |
|  | DNA/CMC-EnSpm | 26,581,953 | 0.55 |
|  | DNA/Dada | 583,661 | 0.01 |
|  | DNA/Ginger | 22,112 | 0 |
|  | DNA/Kolobok-T2 | 321,331 | 0.01 |
|  | DNA/MULE-MuDR | 7,800,958 | 0.16 |
|  | DNA/Maverick | 991,152 | 0.02 |
|  | DNA/Merlin | 303,221 | 0.01 |
|  | DNA/Novosib | 7,346,050 | 0.15 |
|  | DNA/P | 3,438,821 | 0.07 |
|  | DNA/PIF-Harbinger | 7,677,085 | 0.16 |
|  | DNA/PIF-ISL2EU | 342,981 | 0.01 |
|  | DNA/PiggyBac | 25,107,004 | 0.52 |
|  | DNA/Sola | 172,819 | 0 |
|  | DNA/TcMar | 5,110,104 | 0.11 |
|  | DNA/TcMar-ISRm11 | 99,506 | 0 |
|  | DNA/TcMar-Tc1 | 2,221,145 | 0.05 |
|  | DNA/TcMar-Tc2 | 264,008 | 0.01 |
|  | DNA/TcMar-Tigger | 9,405,795 | 0.2 |
|  | DNA/TcMar-m44 | 1,018,166 | 0.02 |
|  | DNA/hAT | 398,308 | 0.01 |
|  | DNA/hAT-Ac | 344,234 | 0.01 |
|  | DNA/hAT-Charlie | 10,071,040 | 0.21 |
|  | DNA/hAT-Tip100 | 2,598,129 | 0.05 |
|  | DNA/others | 15,582,284 | 0.32 |
| LINE | LINE/CR1 | 276,216,274 | 5.75 |
|  | LINE/CR1-Zenon | 216,478,050 | 4.51 |
|  | LINE/CRE | 2,930,286 | 0.06 |
|  | LINE/CRE-II | 10,819,796 | 0.23 |
|  | LINE/DRE | 266,152 | 0.01 |
|  | LINE/I | 18,482,701 | 0.38 |
|  | LINE/I-Nimb | 5,364,514 | 0.11 |
|  | LINE/Jockey | 74,442,264 | 1.55 |
|  | LINE/L1 | 11,755,551 | 0.24 |
|  | LINE/L2 | 26,210,725 | 0.55 |
|  | LINE/Penelope | 50,732,174 | 1.06 |
|  | LINE/Proto2 | 14,193,403 | 0.3 |
|  | LINE/RTE-BovB | 195,709,681 | 4.07 |
|  | LINE/RTE-RTE | 1,519,461 | 0.03 |
|  | LINE/RTE-X | 3,720,561 | 0.08 |
|  | LINE/Rex-Babar | 788,910 | 0.02 |
|  | LINE/others | 2,021,941 | 0.04 |
| LTR | LTR/Copia | 14,717,354 | 0.31 |
|  | LTR/DIRS | 144,349,973 | 3 |
|  | LTR/ERV1 | 15,714,707 | 0.33 |
|  | LTR/Gypsy | 255,066,689 | 5.31 |
|  | LTR/Gypsy-Cigr | 2,319,713 | 0.05 |
|  | LTR/Pao | 68,216,590 | 1.42 |
| SINE | SINE/ID | 582,462 | 0.01 |
|  | SINE/MIR | 148,530 | 0 |
|  | SINE/tRNA | 2,608,250 | 0.05 |
|  | SINE/tRNA-Deu-L2 | 1,243,492 | 0.03 |
|  | SINE/tRNA-L2 | 1,593,644 | 0.03 |
|  | SINE/tRNA-RTE | 1,000,022 | 0.02 |
|  | SINE/others | 2,241,239 | 0.05 |
| Unknown | Unknown | 1,487,300,190 | 30.95 |

**Table S12. Statistics of the gene family among species.**

| Species | Gene number | Gene in family | Unclustered genes | Family number | Unique families |
| --- | --- | --- | --- | --- | --- |
| *D. magna* | 26,646 | 20,662 | 5,984 | 7,650 | 445 |
| *D. pulex* | 30,634 | 26,564 | 4,070 | 7,936 | 760 |
| *E. affinis* | 20,716 | 16,332 | 4,384 | 6,154 | 531 |
| *H. azteca* | 12,906 | 11,990 | 916 | 4,567 | 70 |
| *P. platypus* | 28,287 | 22,717 | 5,570 | 6,286 | 734 |
| *D. melanogaster* | 13,931 | 11,791 | 2,140 | 5,779 | 242 |
| *B. anynana* | 14,413 | 13,034 | 1,379 | 5,894 | 228 |
| *B. terrestris* | 10,581 | 9,419 | 1,162 | 5,641 | 46 |
| *E. j. sinensis* | 22,619 | 21,207 | 1,412 | 7,505 | 185 |
| *P. trituberculatus* | 16,796 | 16,178 | 618 | 6,861 | 64 |
| *A. aegypti* | 14,626 | 13,642 | 984 | 5,993 | 265 |
| *P. vannamei* | 25,527 | 18,837 | 6,690 | 6,849 | 315 |
| *S. mimosarum* | 27,135 | 20,721 | 6,414 | 5,861 | 398 |

**Table S13. The relative evolution rate of species employed by LINTRE.**

| Outgroup | Ingroup1 | Ingroup2 | bA | bB | delta | Z score | CP |
| --- | --- | --- | --- | --- | --- | --- | --- |
| *S. mimosarum* | *P. platypus* | *E. j. sinensis* | 0.186905 | 0.208994 | 0.022089 | 2.326546 | 97.96% |
| *S. mimosarum* | *P. platypus* | *P. trituberculatus* | 0.16443 | 0.216615 | 0.052184 | 5.658374 | 99.96% |
| *S. mimosarum* | *P. platypus* | *A. aegypti* | 0.506799 | 0.535023 | 0.028224 | 2.075423 | 96.16% |
| *S. mimosarum* | *P. platypus* | *P. vannamei* | 0.140617 | 0.230192 | 0.089574 | 9.727365 | 99.96% |
| *S. mimosarum* | *P. platypus* | *D. magna* | 0.462578 | 0.527831 | 0.065253 | 4.904058 | 99.96% |
| *S. mimosarum* | *P. platypus* | *D. pulex* | 0.443761 | 0.511343 | 0.067581 | 5.229217 | 99.96% |
| *S. mimosarum* | *P. platypus* | *E. affinis* | 0.58361 | 0.496107 | 0.087502 | 5.950127 | 99.96% |
| *S. mimosarum* | *P. platypus* | *H. azteca* | 0.508979 | 0.313427 | 0.195552 | 13.04331 | 99.96% |
| *S. mimosarum* | *P. platypus* | *D. melanogaster* | 0.593807 | 0.528602 | 0.065205 | 4.550195 | 99.96% |
| *S. mimosarum* | *P. platypus* | *B. anynana* | 0.541237 | 0.541611 | 0.000374 | 0.026904 | 1.60% |
| *S. mimosarum* | *P. platypus* | *B. terrestris* | 0.445532 | 0.550612 | 0.10508 | 8.049642 | 99.96% |

Note: The *P. platypus* was used as the reference species, and *S. mimosarum* was used as an outgroup species. Z-statistic was used to test whether the distances between ingroups (bA, bB) to outgroup has significant differences. Delta is the absolute difference between bA and bB (delta = | bA - bB |). Z-statistics (Z score) is calculated with the formula of Z = delta/s.e., where delta represents the absolute difference between bA and bB, and s.e represents the standard error; CP (confident probability) is equal to 1-*P*-value (CP = 1 - *P*-value).

**Table S14. The relative evolution rate of species employed by MEGA.**

| Outgroup | Ingroup1 | Ingroup2 | Identical site | Ingroup1 specific | Ingroup2 specific | Chi-score | *P*-value |
| --- | --- | --- | --- | --- | --- | --- | --- |
| *S. mimosarum* | *P. platypus* | *D. magna* | 8,991 | 2,833 | 2,475 | 24.15 | 0.00000 |
| *S. mimosarum* | *P. platypus* | *D. pulex* | 9,045 | 2,790 | 2,412 | 27.47 | 0.00000 |
| *S. mimosarum* | *P. platypus* | *E. affinis* | 8,364 | 2,438 | 2,873 | 35.63 | 0.00000 |
| *S. mimosarum* | *P. platypus* | *H. azteca* | 7,557 | 1,504 | 2,326 | 176.42 | 0.00000 |
| *S. mimosarum* | *P. platypus* | *D. melanogaster* | 8,748 | 2,596 | 2,935 | 20.78 | 0.00001 |
| *S. mimosarum* | *P. platypus* | *B. anynana* | 8,811 | 2,764 | 2,762 | 0.00 | 0.97854 |
| *S. mimosarum* | *P. platypus* | *B. terrestris* | 9,272 | 3,001 | 2,406 | 65.48 | 0.00000 |
| *S. mimosarum* | *P. platypus* | *E. j. sinensis* | 10,056 | 1,278 | 1,163 | 5.42 | 0.01993 |
| *S. mimosarum* | *P. platypus* | *P. trituberculatus* | 10,046 | 1,285 | 1,013 | 32.19 | 0.00000 |
| *S. mimosarum* | *P. platypus* | *A. aegypti* | 9,009 | 2,828 | 2,674 | 4.31 | 0.03788 |
| *S. mimosarum* | *P. platypus* | *P. vannamei* | 10,135 | 1,427 | 949 | 96.16 | 0.00000 |

Note: The *P. platypus* was used as the reference species, and *S. mimosarum* was used as an outgroup species. The “Identical site” represents the number of identical sites observed among groups (outgroup, A species, and B species). The “Ingroup1 specific” represents the number of specific sites observed in group A, and “Ingroup2 specific” represents the number of specific sites observed in group B. Chi-score represents the statistic value calculating from the [Chi-Square](http://www.baidu.com/link?url=F6PLtRejD6rOffPL7dC-RIUXZV1YNqyBZSWf5H5QGaiR5vqKOs5UNbN2TnQSKEZfRbRljHx7gY0Ta-UrIb9GAWqU8COSLk4QIY4Zs_57STW) test.

**Table S15. The GO enrichment analysis of common gene families of all Malacostraca species we used.**

| GO ID | GO Term | GO Class | *P*-value |
| --- | --- | --- | --- |
| GO:0005515 | protein binding | MF | 7.05e-10 |
| GO:0044238 | primary metabolic process | BP | 2.11e-04 |
| GO:0071704 | organic substance metabolic process | BP | 2.11e-04 |
| GO:0008146 | sulfotransferase activity | MF | 4.70e-04 |
| GO:0016782 | transferase activity, transferring sulfur-containing groups | MF | 4.70e-04 |
| GO:0005975 | carbohydrate metabolic process | BP | 6.45e-04 |
| GO:0008152 | metabolic process | BP | 8.62e-04 |
| GO:0016874 | ligase activity | MF | 1.21e-02 |
| GO:0005921 | gap junction | CC | 1.31e-02 |
| GO:0005576 | extracellular region | CC | 1.31e-02 |
| GO:0005911 | cell-cell junction | CC | 1.31e-02 |
| GO:0030054 | cell junction | CC | 1.31e-02 |
| GO:0016020 | membrane | CC | 1.82e-02 |
| GO:0005575 | cellular_component | CC | 1.92e-02 |
| GO:0004842 | ubiquitin-protein ligase activity | MF | 3.63e-02 |
| GO:0006629 | lipid metabolic process | BP | 3.63e-02 |
| GO:0016879 | ligase activity, forming carbon-nitrogen bonds | MF | 3.63e-02 |
| GO:0016881 | acid-amino acid ligase activity | MF | 3.63e-02 |
| GO:0019787 | small conjugating protein ligase activity | MF | 3.63e-02 |
| GO:0044765 | single-organism transport | BP | 4.93e-02 |

**Table S16. The KEGG enrichment analysis of common gene families of all Malacostraca species we used.**

| Map ID | Pathway | Count | *P*-value |
| --- | --- | --- | --- |
| map00480 | Glutathione metabolism | 93 | 6.10e-06 |
| map00980 | Metabolism of xenobiotics by cytochrome P450 | 73 | 7.62e-06 |
| map04919 | Thyroid hormone signaling pathway | 80 | 1.47e-05 |
| map00590 | Arachidonic acid metabolism | 47 | 2.33e-05 |
| map00982 | Drug metabolism - cytochrome P450 | 67 | 3.40e-05 |
| map00983 | Drug metabolism - other enzymes | 64 | 7.07e-05 |
| map00600 | Sphingolipid metabolism | 36 | 9.17e-05 |
| map04640 | Hematopoietic cell lineage | 75 | 1.29e-04 |
| map04721 | Synaptic vesicle cycle | 34 | 1.67e-04 |
| map00591 | Linoleic acid metabolism | 40 | 1.71e-04 |
| map02010 | ABC transporters | 43 | 3.33e-04 |
| map00830 | Retinol metabolism | 61 | 4.15e-04 |
| map04726 | Serotonergic synapse | 59 | 6.48e-04 |
| map04918 | Thyroid hormone synthesis | 59 | 6.48e-04 |
| map00140 | Steroid hormone biosynthesis | 63 | 6.88e-04 |
| map04915 | Estrogen signaling pathway | 67 | 7.14e-04 |
| map05120 | Epithelial cell signaling in Helicobacter pylori infection | 44 | 9.11e-04 |
| map00910 | Nitrogen metabolism | 20 | 1.52e-03 |
| map04662 | B cell receptor signaling pathway | 70 | 1.93e-03 |
| map01120 | Microbial metabolism in diverse environments | 184 | 1.93e-03 |
| map00020 | Citrate cycle (TCA cycle) | 31 | 2.04e-03 |
| map05020 | Prion diseases | 40 | 2.40e-03 |
| map04912 | GnRH signaling pathway | 64 | 2.89e-03 |
| map00062 | Fatty acid elongation | 18 | 2.91e-03 |
| map00053 | Ascorbate and aldarate metabolism | 33 | 4.30e-03 |
| map05110 | Vibrio cholerae infection | 41 | 5.21e-03 |
| map00513 | Various types of N-glycan biosynthesis | 45 | 5.38e-03 |
| map00363 | Bisphenol degradation | 22 | 5.65e-03 |
| map04961 | Endocrine and other factor-regulated calcium reabsorption | 27 | 5.88e-03 |
| map00970 | Aminoacyl-tRNA biosynthesis | 36 | 6.12e-03 |
| map04745 | Phototransduction - fly | 40 | 6.48e-03 |
| map05210 | Colorectal cancer | 31 | 7.01e-03 |
| map04340 | Hedgehog signaling pathway | 39 | 8.06e-03 |
| map04668 | TNF signaling pathway | 30 | 8.92e-03 |
| map04310 | Wnt signaling pathway | 79 | 9.43e-03 |
| map04614 | Renin-angiotensin system | 34 | 9.65e-03 |
| map00350 | Tyrosine metabolism | 46 | 9.87e-03 |
| map04370 | VEGF signaling pathway | 38 | 9.98e-03 |
| map04650 | Natural killer cell mediated cytotoxicity | 42 | 1.00e-02 |
| map04120 | Ubiquitin mediated proteolysis | 107 | 1.29e-02 |
| map01212 | Fatty acid metabolism | 52 | 1.32e-02 |
| map00040 | Pentose and glucuronate interconversions | 40 | 1.50e-02 |
| map05161 | Hepatitis B | 58 | 1.63e-02 |
| map00360 | Phenylalanine metabolism | 27 | 1.81e-02 |
| map05323 | Rheumatoid arthritis | 31 | 1.87e-02 |
| map00120 | Primary bile acid biosynthesis | 12 | 2.05e-02 |
| map00430 | Taurine and hypotaurine metabolism | 12 | 2.05e-02 |
| map00604 | Glycosphingolipid biosynthesis - ganglio series | 12 | 2.05e-02 |
| map00930 | Caprolactam degradation | 12 | 2.05e-02 |
| map05145 | Toxoplasmosis | 80 | 2.18e-02 |
| map04510 | Focal adhesion | 171 | 2.22e-02 |
| map04261 | Adrenergic signaling in cardiomyocytes | 56 | 2.26e-02 |
| map05214 | Glioma | 34 | 2.29e-02 |
| map00670 | One carbon pool by folate | 17 | 2.31e-02 |
| map00280 | Valine, leucine and isoleucine degradation | 45 | 2.39e-02 |
| map04660 | T cell receptor signaling pathway | 41 | 2.55e-02 |
| map04966 | Collecting duct acid secretion | 11 | 2.83e-02 |
| map00260 | Glycine, serine and threonine metabolism | 58 | 2.84e-02 |
| map04725 | Cholinergic synapse | 51 | 2.89e-02 |
| map04750 | Inflammatory mediator regulation of TRP channels | 68 | 2.90e-02 |
| map01200 | Carbon metabolism | 97 | 2.98e-02 |
| map04916 | Melanogenesis | 61 | 3.02e-02 |
| map00740 | Riboflavin metabolism | 16 | 3.04e-02 |
| map05032 | Morphine addiction | 47 | 3.13e-02 |
| map00514 | Other types of O-glycan biosynthesis | 32 | 3.42e-02 |
| map04664 | Fc epsilon RI signaling pathway | 32 | 3.42e-02 |
| map01210 | 2-Oxocarboxylic acid metabolism | 20 | 3.46e-02 |
| map02020 | Two-component system | 28 | 3.54e-02 |
| map04390 | Hippo signaling pathway | 66 | 3.83e-02 |
| map04620 | Toll-like receptor signaling pathway | 35 | 3.91e-02 |
| map05416 | Viral myocarditis | 35 | 3.91e-02 |
| map04380 | Osteoclast differentiation | 42 | 4.02e-02 |
| map00410 | beta-Alanine metabolism | 31 | 4.15e-02 |
| map00860 | Porphyrin and chlorophyll metabolism | 38 | 4.35e-02 |
| map05215 | Prostate cancer | 38 | 4.35e-02 |
| map00601 | Glycosphingolipid biosynthesis - lacto and neolacto series | 27 | 4.35e-02 |
| map05213 | Endometrial cancer | 27 | 4.35e-02 |
| map05223 | Non-small cell lung cancer | 27 | 4.35e-02 |
| map01230 | Biosynthesis of amino acids | 65 | 4.38e-02 |
| map04978 | Mineral absorption | 19 | 4.40e-02 |
| map00720 | Carbon fixation pathways in prokaryotes | 23 | 4.47e-02 |
| map04010 | MAPK signaling pathway | 127 | 4.62e-02 |
| map05203 | Viral carcinogenesis | 90 | 4.67e-02 |
| map00620 | Pyruvate metabolism | 34 | 4.69e-02 |
| map04064 | NF-kappa B signaling pathway | 34 | 4.69e-02 |
| map05130 | Pathogenic Escherichia coli infection | 61 | 4.84e-02 |

**Table S17. The GO enrichment analysis of *P. platypus* specific gene families.**

| GO ID | GO Term | GO Class | *P*-value |
| --- | --- | --- | --- |
| GO:0003676 | nucleic acid binding | MF | 4.35e-34 |
| GO:0003677 | DNA binding | MF | 6.10e-34 |
| GO:0097159 | organic cyclic compound binding | MF | 2.46e-23 |
| GO:1901363 | heterocyclic compound binding | MF | 2.46e-23 |
| GO:0016779 | nucleotidyltransferase activity | MF | 2.81e-05 |
| GO:0005085 | guanyl-nucleotide exchange factor activity | MF | 9.33e-04 |
| GO:0005488 | binding | MF | 2.29e-03 |
| GO:0030695 | GTPase regulator activity | MF | 8.79e-03 |
| GO:0060589 | nucleoside-triphosphatase regulator activity | MF | 8.79e-03 |
| GO:0003674 | molecular_function | MF | 1.07e-02 |
| GO:0016772 | transferase activity, transferring phosphorus-containing groups | MF | 1.90e-02 |
| GO:0006413 | translational initiation | BP | 3.07e-02 |

**Table S18. The KEGG enrichment analysis of *P. platypus* specific gene families.**

| Map ID | Pathway | Count | *P*-value |
| --- | --- | --- | --- |
| map04013 | MAPK signaling pathway - fly | 3 | 1.22e-02 |
| map04974 | Protein digestion and absorption | 5 | 2.15e-02 |
| map05142 | Chagas disease (American trypanosomiasis) | 3 | 2.76e-02 |
| map00860 | Porphyrin and chlorophyll metabolism | 3 | 3.30e-02 |
| map04623 | Cytosolic DNA-sensing pathway | 2 | 3.50e-02 |
| map05222 | Small cell lung cancer | 4 | 4.07e-02 |
| map00051 | Fructose and mannose metabolism | 3 | 4.09e-02 |
| map00563 | Glycosylphosphatidylinositol(GPI)-anchor biosynthesis | 2 | 4.21e-02 |
| map05168 | Herpes simplex infection | 5 | 4.32e-02 |

**Table S19. The GO enrichment analysis of expanded gene families in Malacostraca species.**

| GO ID | GO Term | GO Class | *P*-value |
| --- | --- | --- | --- |
| GO:0008146 | sulfotransferase activity | MF | 2.23e-20 |
| GO:0016782 | transferase activity, transferring sulfur-containing groups | MF | 2.23e-20 |
| GO:0005911 | cell-cell junction | CC | 1.90e-12 |
| GO:0005921 | gap junction | CC | 1.90e-12 |
| GO:0030054 | cell junction | CC | 1.90e-12 |
| GO:0016740 | transferase activity | MF | 3.56e-06 |
| GO:0005515 | protein binding | MF | 4.52e-06 |
| GO:0005488 | binding | MF | 1.27e-05 |
| GO:0003676 | nucleic acid binding | MF | 1.39e-05 |
| GO:0003674 | molecular_function | MF | 9.08e-05 |
| GO:0097159 | organic cyclic compound binding | MF | 2.97e-04 |
| GO:1901363 | heterocyclic compound binding | MF | 2.97e-04 |
| GO:0005576 | extracellular region | CC | 4.40e-04 |
| GO:0005525 | GTP binding | MF | 8.03e-04 |
| GO:0019001 | guanyl nucleotide binding | MF | 8.03e-04 |
| GO:0032561 | guanyl ribonucleotide binding | MF | 8.03e-04 |
| GO:0004866 | endopeptidase inhibitor activity | MF | 1.07e-03 |
| GO:0061135 | endopeptidase regulator activity | MF | 1.07e-03 |
| GO:0005615 | extracellular space | CC | 1.16e-03 |
| GO:0044421 | extracellular region part | CC | 1.16e-03 |
| GO:0030414 | peptidase inhibitor activity | MF | 3.18e-03 |
| GO:0061134 | peptidase regulator activity | MF | 3.18e-03 |
| GO:0004857 | enzyme inhibitor activity | MF | 4.98e-03 |
| GO:0005882 | intermediate filament | CC | 1.61e-02 |
| GO:0045095 | keratin filament | CC | 1.61e-02 |
| GO:0045111 | intermediate filament cytoskeleton | CC | 1.61e-02 |

**Table S20. The KEGG enrichment analysis of expanded gene families in Malacostraca species.**

| Map ID | Pathway | Count | *P*-value |
| --- | --- | --- | --- |
| map00591 | Linoleic acid metabolism | 15 | 1.32e-05 |
| map04975 | Fat digestion and absorption | 15 | 1.84e-05 |
| map04340 | Hedgehog signaling pathway | 15 | 2.52e-05 |
| map04150 | mTOR signaling pathway | 17 | 4.09e-05 |
| map03013 | RNA transport | 39 | 5.85e-05 |
| map00240 | Pyrimidine metabolism | 32 | 9.82e-05 |
| map05205 | Proteoglycans in cancer | 39 | 1.04e-04 |
| map04720 | Long-term potentiation | 23 | 1.06e-04 |
| map04726 | Serotonergic synapse | 18 | 1.17e-04 |
| map00740 | Riboflavin metabolism | 8 | 1.57e-04 |
| map00100 | Steroid biosynthesis | 10 | 1.85e-04 |
| map00950 | Isoquinoline alkaloid biosynthesis | 8 | 2.56e-04 |
| map00514 | Other types of O-glycan biosynthesis | 12 | 2.80e-04 |
| map04914 | Progesterone-mediated oocyte maturation | 18 | 3.84e-04 |
| map04724 | Glutamatergic synapse | 31 | 4.21e-04 |
| map03008 | Ribosome biogenesis in eukaryotes | 23 | 5.88e-04 |
| map04913 | Ovarian steroidogenesis | 13 | 8.86e-04 |
| map04977 | Vitamin digestion and absorption | 13 | 8.86e-04 |
| map03018 | RNA degradation | 24 | 1.05e-03 |
| map00532 | Glycosaminoglycan biosynthesis - chondroitin sulfate / dermatan sulfate | 8 | 1.77e-03 |
| map04722 | Neurotrophin signaling pathway | 20 | 2.21e-03 |
| map04750 | Inflammatory mediator regulation of TRP channels | 18 | 2.27e-03 |
| map04114 | Oocyte meiosis | 19 | 3.14e-03 |
| map04918 | Thyroid hormone synthesis | 15 | 3.20e-03 |
| map00230 | Purine metabolism | 42 | 6.13e-03 |
| map05140 | Leishmaniasis | 6 | 2.36e-02 |
| map00983 | Drug metabolism - other enzymes | 13 | 3.08e-02 |
| map03460 | Fanconi anemia pathway | 11 | 3.10e-02 |
| map00140 | Steroid hormone biosynthesis | 13 | 3.41e-02 |

**Table S21. The GO enrichment analysis of contracted gene families in Malacostraca species.**

| GO ID | GO Term | GO Class | *P*-value |
| --- | --- | --- | --- |
| GO:0005515 | protein binding | MF | 2.49e-05 |
| GO:0005488 | binding | MF | 1.01e-02 |

**Table S22. The KEGG enrichment analysis of contracted gene families in Malacostraca species.**

| Map ID | Pathway | Count | *P*-value |
| --- | --- | --- | --- |
| map00071 | Fatty acid degradation | 6 | 1.03e-04 |
| map05323 | Rheumatoid arthritis | 5 | 2.10e-04 |
| map01212 | Fatty acid metabolism | 6 | 3.93e-04 |
| map05206 | MicroRNAs in cancer | 8 | 7.72e-04 |
| map04977 | Vitamin digestion and absorption | 5 | 8.54e-04 |
| map04142 | Lysosome | 11 | 2.22e-03 |
| map03320 | PPAR signaling pathway | 3 | 1.53e-02 |

**Table S23. The GO enrichment analysis of expanded gene families in *P. platypus*.**

| GO ID | GO Term | GO Class | *P*-value |
| --- | --- | --- | --- |
| GO:0008146 | sulfotransferase activity | MF | 1.39e-21 |
| GO:0016782 | transferase activity, transferring sulfur-containing groups | MF | 1.39e-21 |
| GO:0003674 | molecular_function | MF | 4.70e-10 |
| GO:0003676 | nucleic acid binding | MF | 7.52e-09 |
| GO:0097159 | organic cyclic compound binding | MF | 6.27e-08 |
| GO:1901363 | heterocyclic compound binding | MF | 6.27e-08 |
| GO:0016740 | transferase activity | MF | 1.79e-07 |
| GO:0005488 | binding | MF | 3.66e-07 |
| GO:0005515 | protein binding | MF | 1.26e-04 |
| GO:0005576 | extracellular region | CC | 2.36e-04 |
| GO:0005525 | GTP binding | MF | 3.21e-04 |
| GO:0019001 | guanyl nucleotide binding | MF | 3.21e-04 |
| GO:0032561 | guanyl ribonucleotide binding | MF | 3.21e-04 |
| GO:0004866 | endopeptidase inhibitor activity | MF | 6.26e-04 |
| GO:0061135 | endopeptidase regulator activity | MF | 6.26e-04 |
| GO:0005615 | extracellular space | CC | 7.90e-04 |
| GO:0044421 | extracellular region part | CC | 7.90e-04 |
| GO:0030414 | peptidase inhibitor activity | MF | 1.90e-03 |
| GO:0061134 | peptidase regulator activity | MF | 1.90e-03 |
| GO:0004857 | enzyme inhibitor activity | MF | 3.01e-03 |

**Table S24. The KEGG enrichment analysis of expanded gene families in *P. platypus*.**

| Map ID | Pathway | Count | *P*-value |
| --- | --- | --- | --- |
| map05144 | Malaria | 15 | 1.07e-05 |
| map00982 | Drug metabolism - cytochrome P450 | 23 | 1.46e-05 |
| map05133 | Pertussis | 16 | 2.13e-05 |
| map00591 | Linoleic acid metabolism | 15 | 9.21e-05 |
| map04150 | mTOR signaling pathway | 18 | 9.24e-05 |
| map04726 | Serotonergic synapse | 20 | 9.72e-05 |
| map03013 | RNA transport | 43 | 1.06e-04 |
| map00100 | Steroid biosynthesis | 11 | 1.40e-04 |
| map04340 | Hedgehog signaling pathway | 15 | 1.69e-04 |
| map04512 | ECM-receptor interaction | 25 | 1.77e-04 |
| map04720 | Long-term potentiation | 25 | 1.77e-04 |
| map00240 | Pyrimidine metabolism | 34 | 4.16e-04 |
| map04146 | Peroxisome | 26 | 5.21e-04 |
| map04914 | Progesterone-mediated oocyte maturation | 19 | 9.99e-04 |
| map00514 | Other types of O-glycan biosynthesis | 12 | 1.24e-03 |
| map04913 | Ovarian steroidogenesis | 13 | 3.92e-03 |
| map03018 | RNA degradation | 25 | 4.45e-03 |
| map03008 | Ribosome biogenesis in eukaryotes | 23 | 5.12e-03 |
| map04724 | Glutamatergic synapse | 31 | 5.81e-03 |
| map03460 | Fanconi anemia pathway | 14 | 7.50e-03 |
| map03440 | Homologous recombination | 16 | 8.22e-03 |
| map04114 | Oocyte meiosis | 20 | 8.52e-03 |
| map00603 | Glycosphingolipid biosynthesis - globo series | 7 | 1.10e-02 |
| map04974 | Protein digestion and absorption | 21 | 1.33e-02 |
| map04918 | Thyroid hormone synthesis | 15 | 1.42e-02 |
| map04151 | PI3K-Akt signaling pathway | 35 | 1.47e-02 |
| map04722 | Neurotrophin signaling pathway | 19 | 2.66e-02 |
| map05152 | Tuberculosis | 15 | 3.77e-02 |
| map05146 | Amoebiasis | 17 | 3.87e-02 |
| map05140 | Leishmaniasis | 6 | 4.78e-02 |
| map04750 | Inflammatory mediator regulation of TRP channels | 16 | 4.91e-02 |

**Table S25. The GO enrichment analysis of contracted gene families in *P. platypus*.**

| GO ID | GO Term | GO Class | *P*-value |
| --- | --- | --- | --- |
| GO:0005911 | cell-cell junction | CC | 5.44e-19 |
| GO:0005921 | gap junction | CC | 5.44e-19 |
| GO:0030054 | cell junction | CC | 5.44e-19 |
| GO:0005515 | protein binding | MF | 6.17e-04 |
| GO:0006508 | proteolysis | BP | 1.25e-03 |
| GO:0004867 | serine-type endopeptidase inhibitor activity | MF | 4.94e-03 |

**Table S26. The KEGG enrichment analysis of contracted gene families in *P. platypus*.**

| Map ID | Pathway | Count | *P*-value |
| --- | --- | --- | --- |
| map00260 | Glycine, serine and threonine metabolism | 13 | 1.84e-05 |
| map04060 | Cytokine-cytokine receptor interaction | 8 | 3.03e-05 |
| map04330 | Notch signaling pathway | 9 | 3.12e-05 |
| map04918 | Thyroid hormone synthesis | 12 | 4.82e-05 |
| map00532 | Glycosaminoglycan biosynthesis - chondroitin sulfate / dermatan sulfate | 7 | 6.18e-05 |
| map00642 | Ethylbenzene degradation | 4 | 6.76e-05 |
| map00601 | Glycosphingolipid biosynthesis - lacto and neolacto series | 8 | 6.80e-05 |
| map00073 | Cutin, suberine and wax biosynthesis | 3 | 1.04e-04 |
| map02010 | ABC transporters | 9 | 2.41e-04 |
| map03040 | Spliceosome | 16 | 4.01e-04 |
| map04630 | Jak-STAT signaling pathway | 8 | 6.47e-04 |
| map04723 | Retrograde endocannabinoid signaling | 9 | 1.61e-03 |
| map05145 | Toxoplasmosis | 12 | 2.06e-03 |
| map05310 | Asthma | 2 | 2.22e-03 |
| map04727 | GABAergic synapse | 8 | 2.42e-03 |
| map04742 | Taste transduction | 4 | 2.48e-03 |
| map04141 | Protein processing in endoplasmic reticulum | 21 | 3.07e-03 |
| map05032 | Morphine addiction | 8 | 4.40e-03 |
| map04144 | Endocytosis | 14 | 4.98e-03 |
| map00362 | Benzoate degradation | 4 | 5.64e-03 |
| map00903 | Limonene and pinene degradation | 4 | 7.10e-03 |
| map04725 | Cholinergic synapse | 8 | 7.44e-03 |
| map05146 | Amoebiasis | 10 | 7.48e-03 |
| map05134 | Legionellosis | 12 | 9.38e-03 |
| map05164 | Influenza A | 12 | 1.14e-02 |
| map00280 | Valine, leucine and isoleucine degradation | 7 | 1.16e-02 |
| map04726 | Serotonergic synapse | 8 | 1.18e-02 |
| map04728 | Dopaminergic synapse | 9 | 1.25e-02 |
| map04930 | Type II diabetes mellitus | 5 | 2.04e-02 |
| map04916 | Melanogenesis | 8 | 2.25e-02 |
| map05323 | Rheumatoid arthritis | 5 | 2.30e-02 |
| map00071 | Fatty acid degradation | 6 | 2.43e-02 |
| map04142 | Lysosome | 19 | 2.94e-02 |
| map04744 | Phototransduction | 3 | 3.10e-02 |
| map00512 | Mucin type O-Glycan biosynthesis | 3 | 4.33e-02 |
| map04640 | Hematopoietic cell lineage | 8 | 4.37e-02 |
| map05202 | Transcriptional misregulation in cancer | 8 | 4.37e-02 |


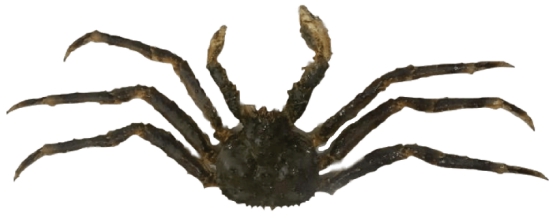


**Figure S1. Blue king crab, *P. platypus*.**


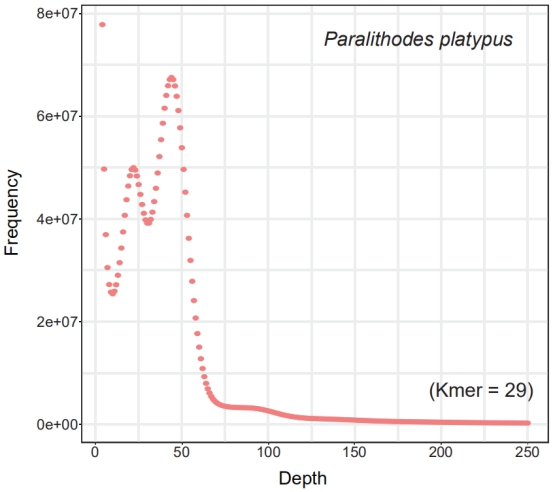


**Figure S2. Genome characteristic analysis of *P. platypus*.**


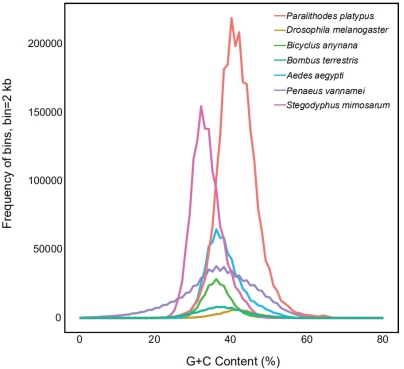


**Figure S3. Analysis of GC distribution in these species.**

Parameters:

1. exonerate: --model est2genome --percent 50 --showtargetgff --showalignment F --showvulgar F --softmasktarget T

2. RAxML: -n orthology -o *S. mimosarum* -m PROTGAMMAAUTO -f a -x 12345 -N 100 -p 12345
